# Use of EST-SSR Markers for Evaluating Genetic Diversity and Fingerprinting Celery (*Apium graveolens* L.) Cultivars

**DOI:** 10.3390/molecules19021939

**Published:** 2014-02-10

**Authors:** Nan Fu, Ping-Yong Wang, Xiao-Dan Liu, Huo-lin Shen

**Affiliations:** College of Agronomy and Biotechnology, China Agricultural University, No.2 Yuanmingyuan Xi Lu, Haidian District, Beijing 100193, China; E-Mails: funan@cau.edu.cn (N.F.); wpy0320fn@cau.edu.cn (P.-Y.W.); liuxiaodan4875527@126.com (X.-D.L.)

**Keywords:** celery, EST-SSR, genetic diversity, fingerprint, genotypic diversity

## Abstract

Celery (*Apium graveolens* L.) is one of the most economically important vegetables worldwide, but genetic and genomic resources supporting celery molecular breeding are quite limited, thus few studies on celery have been conducted so far. In this study we made use of simple sequence repeat (SSR) markers generated from previous celery transcriptome sequencing and attempted to detect the genetic diversity and relationships of commonly used celery accessions and explore the efficiency of the primers used for cultivars identification. Analysis of molecular variance (AMOVA) of *Apium graveolens* L. var. *dulce* showed that approximately 43% of genetic diversity was within accessions, 45% among accessions, and 22% among horticultural types. The neighbor-joining tree generated by unweighted pair group method with arithmetic mean (UPGMA), and population structure analysis, as well as principal components analysis (PCA), separated the cultivars into clusters corresponding to the geographical areas where they originated. Genetic distance analysis suggested that genetic variation within *Apium graveolens* was quite limited. Genotypic diversity showed any combinations of 55 genic SSRs were able to distinguish the genotypes of all 30 accessions.

## 1. Introduction

Celery (*Apium graveolens* L.) is a biennial species from the family of Apiaceae with 2n = 2x = 22 chromosomes. It originated from the Mediterranean basin and several cultivated types are grown worldwide for consumption. Besides the wild (*Apium chilanse*) species and celeriac (*Apium graveolens* L. var. *rapaceum*) species, both coming from Western countries, common celery (*Apium graveolens* L. var. *dulce*) cultivars are generally classification based on their origin as celery (cultivars introduced from Western countries), local celery (Chinese celery) and the middle type (hybrids of celery and local celery).

Several types of biochemical and molecular markers have been applied for celery genotyping, such as isozymes [1], restriction fragment length polymorphism (RFLP) [2], random amplified polymorphic DNA (RAPD) [3,4], amplified fragment length polymorphism (AFLP) [5,6], sequence-related amplified polymorphism (SRAP) [7], expressed sequence tag based SSR (EST-SSR) [8,9]. Genotyping with molecular markers is used for identification of cultivars [10,11], cultivar fingerprinting [12,13], detection of genetic variation and genetic diversity [14,15,16], construction of linkage maps [17,18,19], mapping genes of interest, and for marker assisted selection (MAS) [20,21,22,23]. These researches are frequently carried out with SSR markers for their co-dominant and multi-allelic nature, which makes them more informative than dominant-types of markers. However, development of SSR markers is costly and time consuming and therefore research on celery was quite limited. Next-generation transcriptome sequencing provides an efficient means to develop SSR markers and it has been applied to many organisms [24,25,26]. In our previous work, we developed a set of EST-SSR markers [27] through transcriptome sequencing.

The objectives of the present work were to: (1) test marker polymorphism on a set of celery cultivars; (2) assess the genetic variation existing in the materials used; (3) detect the genetic diversity and population structure of these materials and (4) explore the efficiency of the primers used for cultivar identification.

## 2. Results and Discussion

A list of the samples investigated in this study is given in Table 1. This set of accessions comprised 28 common cultivars, one celeriac and one wild species. The 28 common cultivars can be further divided into 16 celery accessions, nine local celery accessions and three middle type accessions.

**Table 1:** List of the 30 accessions genotyped with genic SSR markers.

Code	Variety	Type	Species
C1	Xuebaiqincai	Local celery	*Apium graveolens* L. var. *dulce*
C3	Jinhuangqincai	Local celery	*Apium graveolens* L. var. *dulce*
C5	Jincuifuqin	Local celery	*Apium graveolens* L. var. *dulce*
C6	Huangxinqin	Local celery	*Apium graveolens* L. var. *dulce*
C8	Shanghaichunqin	Local celery	*Apium graveolens* L. var. *dulce*
C13	Tieganqing	Local celery	*Apium graveolens* L. var. *dulce*
C29	Wuqiangshiganqincai	Local celery	*Apium graveolens* L. var. *dulce*
C37	Shixinqin	Local celery	*Apium graveolens* L. var. *dulce*
C53	Duolunshiganqincai	Local celery	*Apium graveolens* L. var. *dulce*
C111	Lino	Celery	*Apium graveolens* L. var. *dulce*
C67	Jiazhouwang	Celery	*Apium graveolens* L. var. *dulce*
C87	Ventura	Celery	*Apium graveolens* L. var. *dulce*
C114	Huanghou	Celery	*Apium graveolens* L. var. *dulce*
C62	Introduced from USA(fertile)	Celery	*Apium graveolens* L. var. *dulce*
C63	Introduced from USA(sterile )	Celery	*Apium graveolens* L. var. *dulce*
C65	-	Celery	*Apium graveolens* L. var. *dulce*
C68	Jiahuang	Celery	*Apium graveolens* L. var. *dulce*
C74	Dore Golden Spartan	Celery	*Apium graveolens* L. var. *dulce*
C79	Kangnaier	Celery	*Apium graveolens* L. var. *dulce*
C83	Bailixiqin	Celery	*Apium graveolens* L. var. *dulce*
C89	TU52-75	Celery	*Apium graveolens* L. var. *dulce*
C97	Kaifengbolicui	Celery	*Apium graveolens* L. var. *dulce*
C118	Guihe	Celery	*Apium graveolens* L. var. *dulce*
C121	Qianfang	Celery	*Apium graveolens* L. var. *dulce*
C123	Ventura (yellow mutant)	Celery	*Apium graveolens* L. var. *dulce*
C58	Majiagouqincai	Middle type	*Apium graveolens* L. var. *dulce*
C99	Jinnanhuangxinqin	Middle type	*Apium graveolens* L. var. *dulce*
C159	Jinnanshiqin	Middle type	*Apium graveolens* L. var. *dulce*
C163	-	Celeriac	*Apium graveolens* L. var. *rapaceum*
C130	-	Wild	*Apium chilanse*

### 2.1. Marker Development

In the previous work [27] we mined approximately 3,000 SSRs using the MISA software. Among the SSRs, mononucleotide motifs were discarded since it was difficult to distinguish genuine mononucleotide repeats from those generated by polyadenylation products, base mismatches or sequencing errors. In present research, we randomly selected 140 SSRs for polymorphism analysis (Table 2).

**Table 2:** SSR markers used in this study.

Primer Name	Forward Primer	Reverse Primer
Fn1	GCGCTTGGTGTATCTCCACT	AGTGCGTCGAAATATCGCTT
Fn2	GCTTCCGCTGTGTATTTTGA	GGGAAGAAACTGCAACTTGG
Fn3	TGAGCTCCACCAACTGACAC	GCATGAGCAGTTCCAAGACA
Fn4	AATTTACCGCTCTTAGCCTCG	ATAGGCAGAATTTGCGACGA
Fn5	TGAAACCCAAGATCACCCAT	TCATATTGACAGGCAACCGA
Fn6	CCAATCTGGGACTGTGTAAGC	TTCCTGGAGGTGAAGGACTG
Fn7	TGGTGTTGCAGTGTGAATCC	ACCGAAGCATCCTTGAACAG
Fn8	TGGTGTTGCAGTGTGAATCC	ACCGAAGCATCCTTGAACAG
Fn9	CATAGGCTAACGCAGCTTCC	AGTACTCCTTCAGCCGACGA
Fn10	CAGGAGGCTGCAATAACACA	GAGTCGCCGGAATATCAAGA
Fn11	CACACAGACGACTGCTGCTT	ACCATGCATGCTCAACTGAT
Fn12	CACACAGACGACTGCTGCTT	ACCATGCATGCTCAACTGAT
Fn13	CGATCAGGGTACTTGGCAAT	TTTCTATATCCGTCTCATTTCTGTT
Fn14	AACCCTAGCTGCCTCTCTCC	CCATGCCACGAATAGCATTA
Fn15	TGTGTTCTCGCATCTCCAAC	CCAATCTCAACATCGCACAG
Fn16	GTTGGTCAATGCTGCTTCCT	TGTGCCAGGGATACCTTCTC
Fn17	TCACTCACTCCCTTGAGCCT	TGAATCAACACCGTCCATTG
Fn18	CAACCTGAACATCGTTGGTG	TCAACTTGATCTCACGGCAG
Fn19	CTCATACGGTCCAGATCGGT	ATGTCCTGGTGAAGGAGGTG
Fn20	GAGATTGCGATAATGGTGGC	CGCATCACATCACTTAACGG
Fn21	CTGCTCTGAAAGGCTCTGCT	ACAGCTGACATCCTTACCGC
Fn22	TTCACTTGTTCAGCGAGACG	CCTAACCCTAGCTCGTCGTG
Fn23	TCCCATCTCCAATTCCAATC	TTCCTTGCAAGACCATAGGC
Fn24	CATGTCACTGTCGAAGCACC	TGACAATTGCCATTCTCCTG
Fn25	GCCTGAGCATCATAAGAGCC	TATTCACCTTCGTATCCGGC
Fn26	TGTTCCATTATGTGTTGCAGTG	GCGAGATGATGTCAGAACGA
Fn27	ACCATGTCCACCACCTTCTC	GCTGGTTATGGTGGTGCTG
Fn28	ATCGCCAACACCTTCTCAAG	AAGGGTGATTCTGATGGTGC
Fn29	CATATCCAGCACCTCCACCT	TCCAATGGCAACTCACAGAG
Fn30	CTCATTCTTCTTCTTGGCCG	TGCTGAAACGCTACCTCCTT
Fn31	ACATGGAATCTTTCACCTTCA	CATGGCCTAGGAGGAGACAA
Fn32	TTCCTGTCCAGCAGTATCCC	GAATTGAGGGGTGAAAAAGC
Fn33	AAATGAGGTGGTGGTGGAAG	CAATGGGTATGGAATCAGGC
Fn34	AGTACGGTGTCTACGACGGG	CCTCCACCATGATTACCACC
Fn35	CATACTTCTTTGGGGGCTCA	ACACAAACTTTCGGCCAGAT
Fn36	AAGGTCAAGGTCCTGTGGTG	GGTTTAGGCCTCCAATAGCC
Fn37	ACAGTACGTGTCTCCCCCTG	AACAACCCTATGATGGCTGC
Fn38	GTTTGAGCCTCCGCTTACAG	TGCCAGTGACACTCTTCACC
Fn39	GTGACGAAGGAATTGACCGT	ATTTGTTGTCGGGTTCCAAA
Fn40	CTGGCACTTGTACGAAACCA	TATGGGCTGTTGATGACAGG
Fn41	TTCAACCCAGACTTCAACCC	GCAGCCTTCAAATCCAGTTC
Fn42	CCCAGCCCTATCAATCTTCA	CCCCTGCCAAGTCTGTTAAT
Fn43	GAGACAGAGACCATGGGGAA	CGGTTTCGGTTTCGATTTTA
Fn44	TCTTGTCCATTAAAAATGTACCCA	TGCGCATAATGAAAGGATCA
Fn45	AGCAGCACAACAACACTTGG	TTAGGGTCTCTTGTCGAGGC
Fn46	GCAAGTTACCACCCCAGAAA	CCTTTTTCAAAAGCTTCCCC
Fn47	AATTGGCCAGAGCAGAGAAA	TCCTTTATCCCTGACCATGC
Fn48	AATGAGGTTGTTTTTCCCCC	GTAAAGGCCCAAACTCCTCC
Fn49	ACAACAATTTCAGGGCCAAG	TCTTGATATCGGCTTCCTGG
Fn50	ACATTTGTTGCTAGGGTGGC	GCACGAATAGCCGTCCTAAA
Fn51	TCCAATCTCCGGAGCAATAC	GCGGTGGACGAGTAAAAGAG
Fn52	AAACCACCAAACAAGGTGCT	TGAAGTGGAGGAGCAGGAGT
Fn53	ATTCCCAGATGGCTGCATAG	AATTCCAGCAAGCTCAAGGA
Fn54	CCCTCTCCCTATCTTCCTCG	TGAGATTGACTCGGTTGCTG
Fn55	AAAGAAGAAACGGGGATGCT	AACAGCAAGCAGTTCAGTAGTCA
Fn56	CTACACCGCCAATTCAACCT	TAAGCATACACCCCCTCACC
Fn57	TAATGGTGGAAAGAAAGGCG	GGCATACCACTCATTTGGCT
Fn58	CCTAGGCGAACTCTCCTCCT	GGGAATGATCCTCCTTCCAT
Fn59	AGAGGGATAAAATCCCGGTG	TGGAAATGCAAAAGAAGCAA
Fn60	CCACCACCACAACTACAACG	AAGCCGAGTAATGCTGGAAA
Fn61	AGGGTTCGTCGGTTGTAGTG	TCCCCATGTCTATTCTTCGC
Fn62	TCTCGACGAGTTTTCACCTG	GGTCTCTTTGGTGCCATTGT
Fn63	TCAATTAGATCCAGACGCCC	TCTTCTGCCTCCTCTTCGAG
Fn64	CTGTTTTCTCCCTCGTCTGC	CCCCATCTCTGCTATAGCTCC
Fn65	CTTTCTGGGTAAGAAGGCCC	CCAACCCACAACGTCTTACC
Fn66	TCATTGGACAAACAGGACGA	CTGTTTGGCGCTCAATTTTT
Fn67	TACATTTGTGGGATTGGGGT	CCGCCAAAACATTGACAGTA
Fn68	TCAGCTAAGCCACCCTGATT	GTTGCTGCTGGAGAAAGGAG
Fn69	TCCATGAATCTTTCAAGCCC	TCCAAAGTCCAATCCCATTT
Fn70	TAACTGAGTCGGTTGGGTCC	CCTCTCTTTTACCAGCCAAGC
Fn71	TCAACACTCAATTTAAACACCCA	TGATAACGATCGTGACGGAA
Fn72	CATCAACACTCGAAATCGAAA	CAAGATGCTTGTTATCCTTGCT
Fn73	GGATCGGAGGAAGGAAGAAA	GGAGGTGGAGGAGGAGAGTT
Fn74	CCCCCAAACAATAAGTATCCC	TTGGAACTTTTTGTGTCCATTG
Fn75	GCCAGCAGTGTCCCATATTT	GCCCGGAAAAATAACAATCA
Fn76	TTCTACCACTTTCCTTGAATCC	CAGGAGCAGTCTCGATTTCC
Fn77	GGTGTATTTGAATATTAACACCTTTCG	AGAGATGGTGGTCTTGTGGG
Fn78	ACAAGCCCCCTCTACTTTGG	ATGTTGCCAGTTCAGGTTCC
Fn79	TGGGACCCATTTCTTGATTT	AAAATTGCTCCGATTTGTGC
Fn80	CCAGGTAAGCCCAGTCTCAA	TTTTTCTCAATTAAAACTTGCTCATTT
Fn81	AATCCTTGAACTAACCGGGG	CTCTTCGCCACCAGATTCAT
Fn82	GGACGCCCAAGAGAGGTAGT	AGTGGTCTCGACATTTTCCC
Fn83	CCACACCTTGATCGTTGAGA	TTGCTTCTTCCGGCTCTTTA
Fn84	GTTACTTGACGGCACCGTTT	ATCAGTTCTTCATCCGTGGG
Fn85	TCACCCTCTCATCCACATCA	GCAGTGGGTGGATCTAGGAA
Fn86	TCAAATGGACGACGAATCAA	TGCAATGATTTATCCCCCTC
Fn87	CACACACAGGACACACATATTTC	GTAAAGCCGTCTTGGACGAG
Fn88	CGGCATCTTCTCTTCCTCAC	TGTTTGGATCTTTTCTGTTTTCA
Fn89	CAGAAGCGGCTCCTTCTCTA	CCCATTTGAGCTTCACCACT
Fn90	CTAAACGACGCCGTTACCAT	GCTTCTCTCCGCCTTGTATG
Fn91	GGCATACATCGGACGCTAAT	TTGACCCTTTATCTCAATACACACA
Fn92	CCCTCTCTCTCCCTCCTGTC	ACGATTAGCCATTGGTGAGC
Fn93	TGTGTGCTGATTTGAAACCC	ACCGACACTCCACCTTCATC
Fn94	CACCTCTGCTTTCACGGAAC	GTCCAAGAGTGGTCCTCACC
Fn95	ATGGTAACACCACCCTGGAA	GCTTCAACCAGGCAAAGACT
Fn96	TACTTACACCCCTCCCTCCC	TGCAGCACAAGGGATTCATA
Fn97	AAGAGCGATCAAGAACAGGG	TCCCATCTCTCTCCCTCGTA
Fn98	TGCGAACAATACAGTCCCAA	CAGATCCAAACACAGAATTAGCA
Fn99	GAAGAAAGAGGAGAAGGCCG	TCTCGAAACCACCCATCTTC
Fn100	GCGATCCCTAATCAATCCAA	CTTTGAGAGTTACGACGGGC
Fn101	TCAATGGTGTAGAACCAGAACAA	CCCAGATGCTTAAAAGAACCA
Fn102	ACAGGAGGCACTGGTCTCAC	CATTAAAATCCCACAAAAACTTCA
Fn103	TCCCATTCCATTTCAACCTC	AGAGGTGTGGGGAGATTGTG
Fn104	GCGGGGACACTCCACTACT	TTGATCATCAGCAGACTGGC
Fn105	CAAAAATTTAACCCCATACCC	ACATGTACGGACGTTGTGGA
Fn106	GCTTTGCACACACACACACA	CTTTCCCTCGACCTCATCCT
Fn107	GATTTTTCCGATGCAGCACT	GGCATGCACCAAACGTTATC
Fn108	GAGGAGGCTGTTACGTGGAG	TCCCTTTTCTCACTCCATTCC
Fn109	GTAGAAGGCCTGCAGATGCT	GTCTTTGCTTCTCCTCACCG
Fn110	GCACCAGCAAGAGGAGACTT	TTGTTGCTTGCCAGTGAAAC
Fn111	AAGCGAGTAGCTGAAAGGCA	CACTACCACCTCCGATTGCT
Fn112	CCAAGCTTCGACCATTGTTT	TTGTACATCGGTGAAACGGA
Fn113	AGCAGAAAGGCGTTCCACTA	GTTGAGCCCTTCCTGCATAA
Fn114	CGCCCTCTTCCTTTATCTCC	CACTTAGGTTTACCGCTGCC
Fn115	CACGTTTGGTGACATTCCAG	ACAATTATCTCCTTCCGCCC
Fn116	TCCTCTCCTCACCAAACCAC	CACAACCCTTCAACATCACG
Fn117	GTGGTTGGTGGGGATCATAG	GCCCAAAGTTCTTCCAAACA
Fn118	CAATCATATTAATCATCCCCAAA	GAGTTGGTCTGCAGGAGGAG
Fn119	TAAGATGCATGAGGCACGAC	GACTTTGATGCGCACTTTCA
Fn120	TGATTTGTGCACCAAAAGGA	GGAGAGTCGACCGATTCAGA
Fn121	AACTCAGCAACCGGAATCAC	ATACGTAGACGCATCGGAGG
Fn122	GCACACAATAAGCCTCCCAT	CACATGCTACAAAACAGGCG
Fn123	CCACTGGACATTTCTTGGCT	TTTACAAGCCCCAACAGAGC
Fn124	CTGGAACCGGAGTAGGTGAA	AACAGCCTTTACCCTTCATCA
Fn125	ATCTGCCTGTAGCCGAACAG	CTCTTAGTTGGCGCTGCTCT
Fn126	ATAATTTGCCCAACGCTCTG	CTCTCTTGAAAAACACGGGC
Fn127	CAACACAAACACCAAAACCCT	CGTGCCTCATTGGGTTCTAT
Fn128	GTTGTACTTGGTGCGGAGGT	CAAAATTCCAAAAGCCCAAA
Fn129	TCTTTCGATTTGGATTTGGG	TAGAGCTCTCGGCCTCTTCA
Fn130	TTGGTGCCATTGTTGTTGTT	AACGCCTTTCTTCCCAATTT
Fn131	CACCGCGATTCTTCTCTCTC	CGACATCGTCTCTCTCCCTC
Fn132	TTCTTTTTCTGTTCCGCCC	CCGCCGTTAGAGACAAACTC
Fn133	ATTGAAACCCCACCACTGAA	AACGGCCAGAAAAAGCTGTA
Fn134	TGGTTGGGGGAGAATTGTAA	TGAGTTTGCCACAACTGACA
Fn135	TCCCGATAACAAGAGAGAGACT	TGGAGATGAACAAGGGAAGG
Fn136	AGTCCTCAGTTCTCCTGGCA	CAGAATGGTGATGCTGATGG
Fn137	CCAGGACATACATACGTTCTCAA	GACGGACTTAGCCCCCTTAT
Fn138	TAGCTGCGGTTGATTCAGTG	ATTATCAGCGGAAGGCACAC
Fn139	TGCACCACCAAAAACACCTA	GAGGAGGGGTTGAGTGATGA
Fn140	TCACCACCCCTAATTACCGA	AGATAAACCGGGGAGCTTGT

### 2.2. Marker Informative Analysis of Accessions

When the 140 developed SSR markers were used for genotyping the set of 30 accessions, 23 markers had no clear bands or were amplified only in very few accessions. These markers were excluded from further analyses, reducing the number of good quality markers to 117 (83.57%). Among those good quality markers, 54 were monomorphic and 63 (53.85%) were polymorphic on the 30 accessions. The successful amplification rate and polymorphism rate were similar to those of our previous work (81.25% and 59.57%, respectively) [27], though the set of accessions previously genotyped with EST-SSRs was not identical to the current set of accessions.

**Figure 1:**
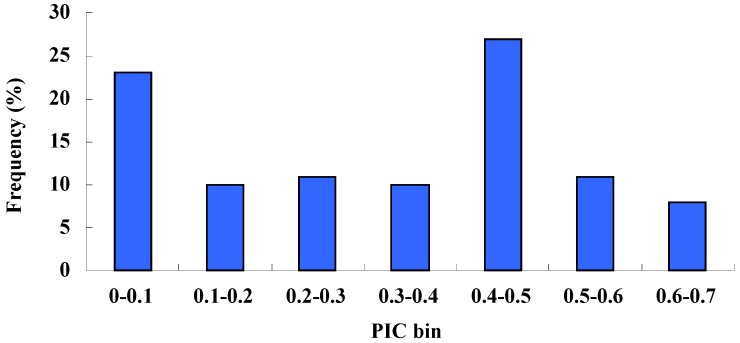
Distribution of polymorphism information content (PIC) values of the SSR markers used for genotyping the 30 studied varieties.

The number of loci per SSR ranged from two to five, with a mean value of 2.71, which was similar to that of previous studies on celery using EST-SSRs (2.68) [27], but lower than the number of ISSR markers reported by Qing-Kuo (5.05) [9]. This indicated that primer sequences designed from SSR flanking regions were highly conserved and SSR markers were more specific than ISSR markers. Polymorphism information content (PIC) values ranged from 0.06 to 0.67, with an average of 0.33. The largest group of markers (27.42%) was in a range from 0.4 to 0.5, followed by the group with PIC values ranging from 0 to 0.1 (Figure 1). The second largest group was made up of markers polymorphic between the wild and cultivated species, but monomorphic within cultivated species.

The number of loci per SSR and the PIC values in our study were low. Generally, it was believed that these indexes for genomic-derived SSRs were significantly higher than EST-SSRs, as indicated by the reports on flax [28], wheat [29], levant cotton [30], sunflower [31] and sugar beet [32]. The lower polymorphism of EST-SSR markers than genomic SSRs was likely due to the conserved nature of genome coding regions [33]. However, it has been reported in some other studies on sorghum [34] and apple [35] that EST-SSR markers have greater discriminating power than the genomic SSRs. The higher average number of alleles per EST-SSR marker reported may be primarily attributed to the difference of species used or the selection of multiple-locus SSRs or compound SSRs, since we usually believe that single-locus SSRs provided less polymorphism. In addition, genotypes may also influence the number of alleles detected at each SSR locus.

**Figure 2:**
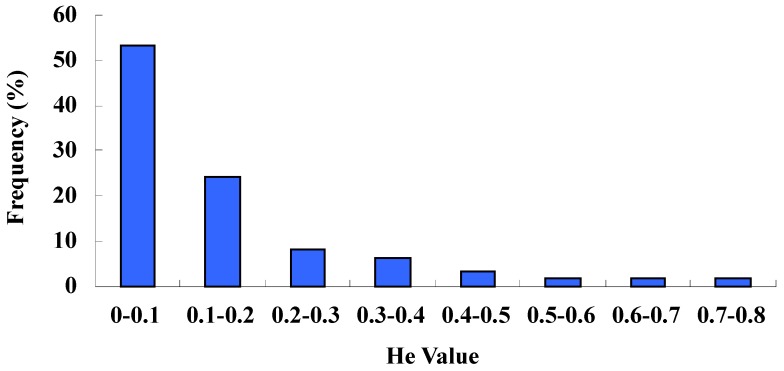
Distribution of the estimate of genetic heterozygosity (*He*).

Based on the polymorphic marker data, we made an analysis of the observed heterozygosity (*Ho*) and expected heterozygosity (*He*). The former varied from 0 to 0.73 (mean 0.13), while the latter varied from 0.07 to 0.68 (mean 0.33). The mean *Ho* and *He* values were similar to the previous results of 0.14 and 0.36, respectively [27]. The distribution of *He* values showed that 85.48% of the markers were in the range from 0 to 0.3, which was a very low heterozygosity (Figure 2). The fact that observed heterozygosity was lower than expected may be due to the small sample size or the results of inbreeding.

### 2.3. Analysis of Molecular Variance (AMOVA)

AMOVA analysis indicated that approximately 35% of the genetic diversity was within individuals, 43% among individuals, and the remaining 22% among horticultural types (Table 3). This was consistent with the findings from other organisms like faba bean [36], grape [37], *Haematococcus pluvialis* [38], olive [39], apple [35] and lettuce [22] showing that considerable genetic diversity was partitioned within, rather than among populations. On the contrary, low levels of genetic diversity within populations and significant genetic differentiation among populations were detected in *Omphalogramma* *souliei*, barely and Chinese-grown pecan [40,41,42].

**Table 3:** Analysis of molecular variance (AMOVA).

Source of variation	Percentage of variation	*p*-value
Among horticultural types	22%	0.001
Among Individuals	43%	0.001
Within Individuals	35%	

We also calculated pairwise differentiation (*F*_st_) for all pairs of horticultural types with at least two accessions per type (Table 4). The variation of the *F*_st_ values ranged from 0.086 to 0.261. Obviously, differentiation between celery and the other two types were significantly higher than that between local celery and the middle type.

**Table 4:** Pairwise differentiation (*F*st) among horticultural types.

Horticultural type	Local celery	Celery	Middle type
Local celery	-		
Celery	0.231	-	
Middle type	0.086	0.261	-

### 2.4. PCA Analysis

**Figure 3:**
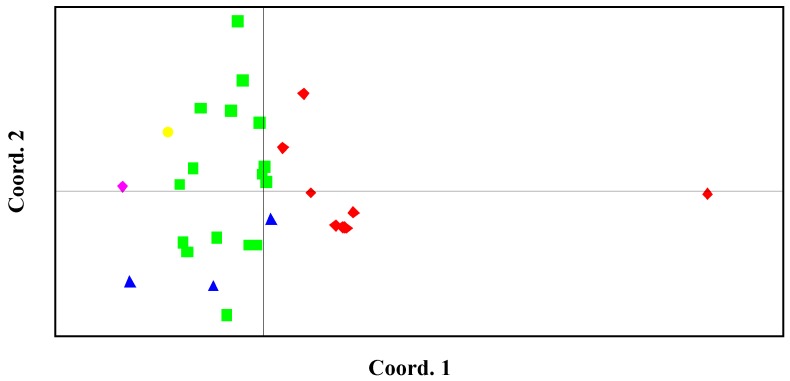
Principal component analysis (PCA) of the 30 accessions genotyped with 62 polymorphic SSRs. Red, local celery; Green, celery; Blue, the middle type; Yellow, celeriac; Pink, the wild type.

The PCA results revealed that accessions of the same horticultural types clustered together. Accessions of local celery were well separated from those of celery. The middle type celery accessions were scattered among celery accessions and were closer to celery cluster than local celery cluster (Figure 3).

PCA analysis unambiguously separated the wild and the var. *rapaceum* (celeriac species) from var. *dulce* (cultivated celery). Compared to the wild species, var. *rapaceum* was closer to var. *dulce*, which may be due to the fact that the studied var. *rapaceum* belonged to cultivars. The observed distances between wild species, celeriac species and var. *dulce* accessions corresponded to the sexual compatibility of the two species with var. *dulce* accessions, which can be further proved by the fact that marker transfer rate was 100% in var. *rapaceum*, but only 54.84% in wild type. What’s more, both celeriac and wild species were the closest to the cluster of celery accessions, but the most distant from the cluster of local celery accessions. This result was supported by the differences in their origins.

### 2.5. Genetic Distance Analysis

**Figure 4:**
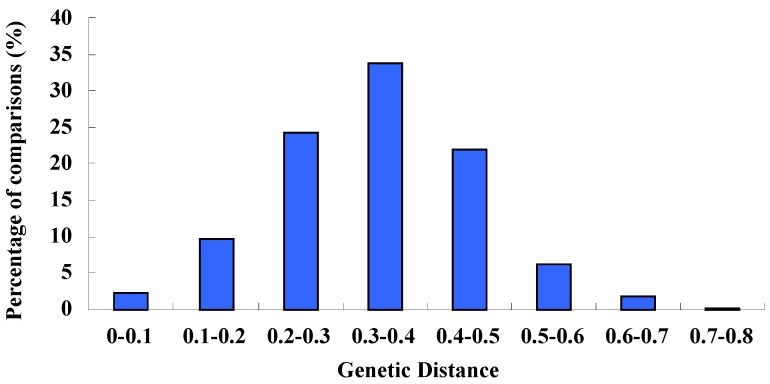
Distribution of genetic distance values obtained from pairwise comparisons of 29 cultivars using SSR marker data.

The average Nei genetic distance for the 29 accessions of var. *dulce* and var. *rapaceum* was 0.34, with a range from 0.26 (C123) to 0.72 (C1). The largest genetic distance (0.72) was between C1 and C83, while the least genetic distance of 0.02 was found between C29 and C97. The average genetic distance of wild species was 2.42 with a range from 2.06 to 3.22, which was much larger than cultivated accessions (less than 0.7). Overall, seventy percent of the genetic distance between any two cultivars was no more than 0.4 and only thirty percent were larger than 0.4 (Figure 4). These results suggested that genetic variation within *Apium graveolens* was limited, while the wild species had wider genetic diversity and could serve as a valuable resource.

### 2.6. Cluster and Population Structure Analysis

**Figure 5:**
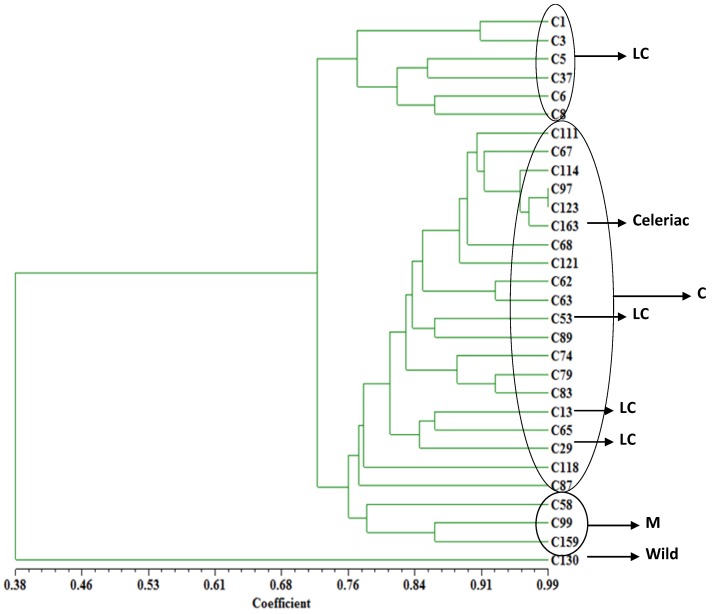
Dendrogram of 30 accessions based on SSR marker data generated from Nei’s genetic distance matrix by UPGMA in NTSYSpc 2.11a. LC: local celery; C: celery; M: middle type.

In order to see the relationship of the materials used in this study, a dendrogram was constructed from the pairwise distance matrices (Figure 5). UPGMA cluster analysis indicated that at the genetic distance of 0.38, cultivated and wild species were separated. The statistical analysis based on the allele frequencies separated most of the cultivars, both in the trees (Figure 5) and in the PCA (Figure 3), into two main clusters corresponding to the geographical areas where they originated. At the distance of 0.72, most local celery accessions formed a cluster and all celery formed a large cluster with three local varieties scattered in. What’s more, the three middle type accessions (C58, C99, and C159) were well clustered together.

We also estimated the number of genetic clusters of 30 accessions using Structure software without specifying prior information concerning sample class and allowing for admixed individuals. In order to choose an appropriate value of *K* for modeling the data, we ran a series of independent runs of the data at a range of values of *K* from 1 to 7.

**Figure 6:**
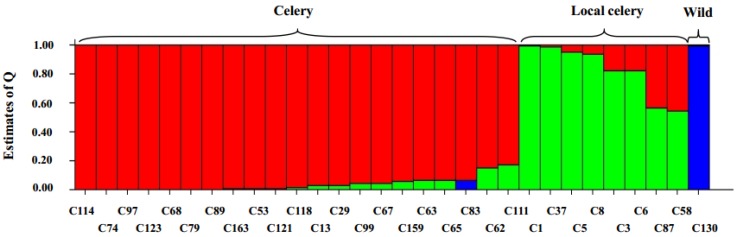
Bar plot of population structure estimates for 30 *Apium* varieties by SSR markers. Each accession is represented by a single vertical bar broken into three colored segments, with lengths proportional to Q of the three inferred populations (*K* = 3). The sum of Q values for each bar is 1. Classes of the materials are shown at the top.

When *K* ranged from 2 to 7, the wild species were separated from the cultivated species and when *K* was larger than 3, the wild species stood alone. When *K* = 3, three populations were obtained (Figure 6).

The first population mainly comprised local celery accessions. The second population contained all celery accessions, the celeriac accessions and three local celery varieties. The third population only included the wild species. This structure was identical with the obtained dendrogram and supported the accuracy of the clustering.

### 2.7. Genotypic Diversity

**Figure 7:**
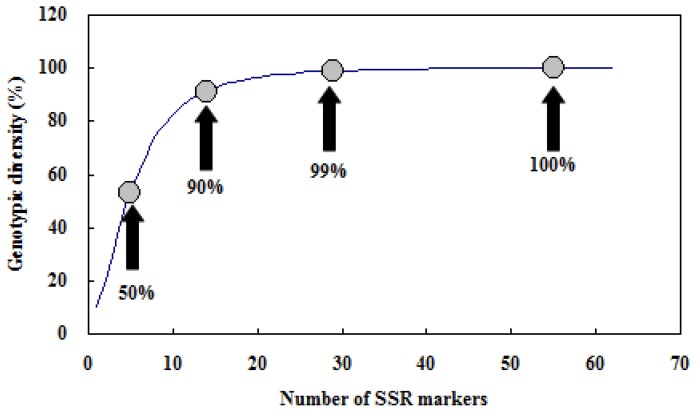
Effect of the increasing number of SSR markers on the estimate of genotyping diversity. Circles indicate genotypic diversity of 50%, 90%, 99%, and 100%, respectively. The value of 100% was reached with 55 and more markers.

Genotypic diversity is defined as the probability that two individuals taken at random have different genotypes. This value is 0 if every individual is the same, and 1 if every individual is different. We used the Multilocus program to calculate the number of different genotypes and the genotypic diversity on the set of 30 accessions. On average five markers were needed to identify 50% of genotypes, 14 markers to identify 90% of genotypes, and 29 markers to identify 99% of genotypes (Figure 7). Our analysis showed that any combinations of 55 SSR markers were able to distinguish genotypes of all 30 accessions unambiguously. This was a relatively high number of markers that were needed for genotyping.

For example, 32 SSR markers were sufficient to distinguish genotypes of all 36 lettuce accessions [22], only 17 SSR markers on average were required to identify 54 sugar beet hybrid varieties [43] and eight SSR markers were enough to distinguish 35 asparagus varieties [44]. In general, genetic similarity among accessions of the same type is high. So it is more difficult to distinguish closely related or less diverse materials. In this study, a total of 50 unique genotypes specific to some accessions were identified by different markers. The number of unique genotype indentified by one primer ranged from 1 to 4. Of these 50 unique genotypes, 23 (46%) exclusively presented in wild species, suggesting the low diversity of the materials used. Therefore more molecular markers were needed to distinguish these closely related materials with high genetic similarity. In addition, the polymorphism of the markers was another important factor affecting whether we can distinguish more genotypes or not. So it is a must to develop higher polymorphic markers to distinguish accessions more efficiently.

## 3. Experimental

### 3.1. Plant Materials and DNA Isolation

A set of 30 accessions (Table 1) was used to test polymorphism of the developed SSR markers. This set comprised 28 common cultivars, one celeriac and one wild species. All materials were grown at the experimental station of China Agricultural University (Beijing, China). Genomic DNA was extracted from celery tender leaves using a modified version of the cetyltrimethylammonium bromide (CTAB) method [45]. Quality of DNA was checked by electrophoresis in 1% agarose gel. The genomic DNA was diluted 10-fold for PCR analysis.

### 3.2. Development of Genic SSR Markers and Genotyping with Markers

The SSR markers were developed through celery transcripotme sequencing [27]. Primers were designed using Primer 3 [46] with default parameters and synthesized at Sangon Biotech Co., Ltd. (Shanghai, China). PCR amplifications were conducted in a final volume of 10 μL containing 3.5 μL 2× Taq PCR MasterMix (Beijing Biomed Co., Ltd., Beijing, China), 4.5 μL double distilled (dd) H_2_O, 0.5 μL of each primer (5 μM) and 1 μL of template (aprox. 20 ng/μL). PCR was performed as follows: denaturation at 94 °C for 5 min, followed by 38 cycles of 30 s at 94 °C, 30 s at Tm (annealing temperature), 1 min at 72 °C and a final step at 72 °C for 10 min. PCR products were firstly detected by agarose gel electrophoresis and the products possessing single band or only a few bands were subjected to 7% polyacrylamide gel to separate alleles. With regard to those had no bands or multiple bands, we optimized the PCR condition to get better products for separation of alleles. PCR products were mixed with a volume of loading buffer and then denatured at 95 °C for 10min before being loaded on the polyacrylamide gel.

### 3.3. Analysis of Marker Polymorphism and Genetic Heterozygosity

SSR alleles were scored manually starting from the smallest to the largest-sized bands. The presence or absence of each single fragment was coded as 1 or 0, respectively, and scored for a binary data matrix. Scored data from polymorphic loci were used to calculate the polymorphism information content (PIC) according to Equation (1):

[PIC = 1 − ∑pi^2^] (1)

where pi is the frequency of ith allele for each locus [47]. Observed heterozygosity (*Ho*) and expected heterozygosity (*He*) were calculated using the Popgene software version 1.31 [48]. *Ho* represents the estimated proportion of observed heterozygotes at a given locus for co-dominant markers. *He*, estimated using the Levene algorithm [49], represents the estimated proportion of expected heterozygotes under random mating for co-dominant markers.

### 3.4. AMOVA and PCA Analysis

Analysis of molecular variance (AMOVA) [50] between all the pairs of horticultural types with at least two accessions, and principal components analysis (PCA) of all accessions were performed using GenAlEx 6.5 [51].

### 3.5. Genetic Diversity and Population Structure Analysis

A genetic similarity matrix was constructed and Nei’s genetic distance [52] was calculated for each pair of all accessions using the NTSYSpc 2.1 software [53]. Unweighted pair group method with arithmetic mean (UPGMA) cluster analysis was performed to develop a dendrogram. Population structure was analyzed using the free software package STRUCTURE 2.3.4 [54,55,56]. A model without prior population information was used to assign individuals to populations.

### 3.6. Identification of Genotypes

In order to see whether scoring more loci is likely to increase the genotypic diversity, or whether one has reached a plateau, we used the software MultiLocus ver. 1.3b [57] to estimate the number of different genotypes that can be identified in a set of 30 accessions with a gradually increasing number of markers. The program randomly sampled from 1 to m−1 loci from the dataset and calculated the number of different genotypes identified.

## 4. Conclusions

This was the first attempts at celery genetic and genotypic diversity analysis using SSR markers developed from transcriptome sequencing. The AMOVA analysis indicated that the largest part of genetic diversity was within populations, while genetic diversity found among populations was low. The geneetic distance of wild species was much larger than that of cultivated accessions, suggesting the wider genetic diversity of the wild species, while the diversity within cultivars was quite limited. PCA analysis revealed that accessions of the same horticultural types were well clustered together. The UPGMA dendrogram and population structure clearly separated wild species from cultivars, and further divided the cultivars into two clusters, corresponding to the geographical areas from where they originated. Genotypic diversity analysis suggested that 29 markers were needed to identify 99% of genotypes and any combinations of 55 SSR markers were able to distinguish genotypes of all 30 accessions. Given that the genetic similarity of commonly used accessions was high, we need to develop more and higher polymorphic markers to efficiently distinguish closely related varieties. This study would provide a common ground for celery accessions identification, breeding and protection of breeders’ rights.
